# Editorial: Reproducibility and rigour in rheumatology

**DOI:** 10.3389/fmed.2024.1402043

**Published:** 2024-03-28

**Authors:** Augusto Silva, João Eurico Fonseca, Stephanie Finzel

**Affiliations:** ^1^Rheumatology and Metabolic Bone Diseases Department, Hospital de Santa Maria, Unidade Local de Saúde de Santa Maria, Lisbon Academic Medical Center, Lisbon, Portugal; ^2^Instituto de Medicina Molecular João Lobo Antunes, Faculdade de Medicina da Universidade de Lisboa, Lisbon, Portugal; ^3^Department of Rheumatology and Clinical Immunology, Medical Center – University of Freiburg, Faculty of Medicine, University of Freiburg, Freiburg im Breisgau, Germany

**Keywords:** scientific studies, reproducibility, misinformation, antinuclear antibody, technological development

Scientific progress in any field of medicine, and rheumatology is no exception, is based on experimentation followed by the publication of accurate and reliable results (1). To achieve these aims, well-designed clinical studies need to be carried out, in such a way that other researchers can replicate them, as well as through a process of strict peer review of scientific journals (1, 2). With the sustained observed expansion of journal publications in the rheumatology field (3), it becomes imperative to ensure the quality of published studies and results. This Research Topic consists of three papers—two review the reproducibility and rigor research, one in antinuclear antibodies (ANA) testing in pediatric practice and the second in general rheumatology field, while the third addresses the health-related misinformation issues on social media.

As research in rheumatology is increasing, rigorous and reproducible studies are essential. Alnaimat et al., in line with other authors, claim that randomized controlled clinical trials (RCTs) are the gold standard for achieving this goal, due to its intrinsic characteristics. However, RCTs have some limitations, such as the very strict inclusion and patient selection criteria, making it difficult to extrapolate to a broader population, as well as the fact that several rheumatic diseases are rare, making it difficult to perform large-scale trials due to technical and time constraints and high costs. It is because of such limitations, real-life studies are often carried out using clinical data from patients, based on medical records. Although these studies have internal validation problems, bias can occur due to treatment indications or practice changes, and missing data is common, affecting the reproducibility and replicability of the results, which might lead to a possible rise of untrustworthy articles being published. Efforts have been made to increase rigor in rheumatology research, such as an automated tool to check adherence to rigor standards (SciScore) and the Rigor and Transparency Index (RTI), a score that rates journals annually based on rigor and transparency. In addition, guidelines of the International Committee of Medical Journal Editors (ICMJE) provide valuable support to authors and editors, while some rheumatology journals request readers access to data and methods without undue restrictions.

A paradigmatic example illustrating the need for rigor and reproducibility applies to the request and the methodology used for ANA detection, respectively. Ostrov et al. review the circumstances for requesting this autoantibody and the methodology used to identify it in pediatric patients. The ANA request is common in clinical practice, even when children have non-specific rheumatological symptoms. However, 70%−90% of these children's symptoms resolve spontaneously, although they may present a false positive ANA test, and clinicians must inform and educate about possible explanations. In addition, there is a high inter-laboratory variability, depending on the methodology used, as well as high intra-laboratory variability in titer and ANA pattern with indirect immunofluorescence (IFA). Consequently, the American College of Rheumatology (ACR) and the American Academy of Pediatrics (AAP) recommend that ANA tests should be only performed by IFA using Hep-2 cells and just be ordered in children with high suspicion or signs of an autoimmune rheumatic disease, such as systemic lupus erythematosus, and therefore their request should be avoided as an autoimmunity screening tool.

The increasing number of studies published other than RCTs raises the risk of misinformation spreading. Polyzou et al. presented a theoretical overview and their opinion about this problem in the field of rheumatology. Technological development in the last few years has also been applied to social networks. On the one hand, it can be a vehicle for misinformation, be it inadvertently or due to other reasons, such as financial or social. On the other hand, it allows a better spread of information among health professionals. The transmission of false information in medicine can be a threat to public health, and this problem has been exacerbated during the COVID-19 pandemic. In order to deal with this situation, health professionals must communicate transparently and effectively, for example through public health campaigns that influence individual behavior. The use of new technologies to share rigorous health information could be an important tool for health professionals to overcome the misinformation issue.

In conclusion, progress in medicine is based on reliable and reproducible studies, ideally through conducting RCTs. Due to the limitations of these studies and the rising number of publications in the rheumatology field, other methodologies have been used, which can lead to less trustworthy results and misinformation as a consequence. Technological progress has increased its applicability in social networks, which can easily transmit misinformation for the public, but can also be used by health professionals to provide credible information to the population, such as the appropriate specifications for requesting ANA and the best methodology for carrying them out, with the IFA being the gold standard. These findings have important implications for health care professionals, researchers, and laboratories and enabling them to provide the best care for the population.

## Author contributions

AS: Conceptualization, Investigation, Writing – original draft. JF: Supervision, Validation, Writing – review & editing. SF: Supervision, Validation, Writing – review & editing.
